# The Beneficial Effects of Professional Identity: The Mediating Role of Teaching Identification in the Relationship between Role Stress and Psychological Distress during the COVID-19 Pandemic

**DOI:** 10.3390/ijerph191811339

**Published:** 2022-09-09

**Authors:** Tyrone Brian Pretorius, Anita Padmanabhanunni

**Affiliations:** Department of Psychology, University of the Western Cape, Bellville 7530, South Africa

**Keywords:** professional identification, role conflict, role ambiguity, anxiety, hopelessness

## Abstract

At the start of the COVID-19 pandemic, teachers and students rapidly transitioned to remote teaching and learning. In South Africa, this initial transition was followed by periods of reopening and closing of schools during the various waves of the pandemic. When schools were reopened, rotational schooling was implemented, with students attending in shifts. All this change created a climate of uncertainty for teachers. The current study investigates the relationship between role stress and indices of psychological distress, as well as the potential mediating role of teaching identification in this relationship, using a cross-sectional survey design. Participants (*n* = 355) were school teachers in South Africa who completed the Role Stress Questionnaire, the Professional Identification Scale, the trait scale of the State-Trait Anxiety Inventory, and the Beck Hopelessness Scale during the second wave of the pandemic (May–July 2021). The results of a structural equation analysis indicate significant positive direct effects of role conflict and ambiguity on anxiety and hopelessness, as well as significant negative direct effects of teaching identification on anxiety and hopelessness. In addition, teaching identification was found to mediate the effect of role conflict and ambiguity on anxiety and hopelessness. The demonstrated role of teaching identification has implications that suggest the importance of leadership and supervisory support, as well as enhancing the societal value of teaching as a profession.

## 1. Introduction

The COVID-19 pandemic has had an unprecedented impact on the education sector globally. One of the primary measures implemented to curb the spread of the virus was school closures. To keep the educational system functional, there was a rapid transition to remote online learning and teaching. Teachers were expected to transform their curriculum to suit online delivery, rapidly acquire skills in information and communication technology (ICT) and guide their students to engage with learning using a new modality [1]. Existing studies, for example [2], have documented the stress experienced by teachers who did not have any prior training in distance education and were required to redesign their curriculum and deliver online courses. Remote online teaching also meant additional work for teachers as it required providing technological support to students and their parents who were not familiar with ICTs and being available in their working roles throughout the day [3].

Remote online teaching impacted teachers’ ability to separate their work life from their personal space. Furthermore, teachers had to manage concurrent responsibilities such as domestic chores, caring for and homeschooling their own children and looking after elderly family members while also planning, preparing and delivering online learning for their students. For many teachers, working from home increased their sense of social isolation and limited contact with supportive resources, including school administrators, managers and their colleagues [3].

Existing studies have reported that teachers experienced the transition to remote online teaching as highly stressful, and this was related to lack of access to technology, doubts regarding their competencies in the use of ICTs, as well as accumulated fatigue, exhaustion and burnout [4]. Prior to the pandemic, teaching was consistently identified as a highly stressful profession associated with an increased risk of burnout and adverse mental health outcomes [5]. The rapid shifts in the expectations of teachers during the COVID-19 pandemic have been identified as a significant source of role stress that is likely to impact teachers’ professional identity and psychological well-being [3].

In role theory, roles are defined as social positions that are associated with normative expectations and behaviors [6]. The concept of roles is key in organizational psychology, and psychologists have proposed that work is lived and experienced through the roles people occupy [7]. Role theory further posits that a major impediment to effective functioning is role stress, which occurs when environmental or social factors negatively impact a person’s ability to meet the perceived requirements of their role. Two aspects of role stress that have been rigorously studied are role conflict and role ambiguity [8]. Role conflict occurs when an individual is given multiple roles at the same time that are different and incompatible, whereas role ambiguity occurs when an individual receives unclear or inconsistent expectations in terms of their role responsibilities [6]. High role conflict and role ambiguity are associated with reduced job satisfaction, increased risk of attrition from the workplace and psychological distress.

The current study was undertaken in South Africa and examined the potential mediating role of teacher identification in the relationship between role stress (i.e., conflict and ambiguity) and indices of psychological distress. As a result of the COVID-19 outbreak, educational institutions in South Africa were closed, and teachers and students had to rapidly transition to remote online teaching and learning. For teachers, the sudden transition to online and remote learning was challenging. This difficult transition, combined with teachers’ anxiety about contracting the virus, balancing home responsibilities while working from home, as well as the impact of the pandemic on the economy, created a very stressful working context. Teachers had to quickly develop new skills to be able to deliver remote teaching, and some teachers had difficulty making this transition. Kraft and colleagues [9] reported that 8% of teachers in their study were uncomfortable with technology. They also found that teachers in different phases of their careers struggled with remote teaching differently. Mid-career teachers found it challenging to balance work responsibilities with home obligations, whereas veteran teachers reported being uncomfortable using technology for remote teaching. All these challenges impacted teachers’ sense of self, what it means to be a teacher, and self-competence. Kraft and colleagues found that 53% of teachers in their study reported that their sense of success decreased during the pandemic [9].

In managing role stress, professional identity can serve as an important resource. Professional identity is a form of social identity that is defined as “one’s professional self-concept based on attributes, beliefs, values, motives, and experiences” [10] (p. 86). It pertains to how teachers perceive themselves based on their ongoing interactions with their work context [11]. The nature of this interaction can lead to job satisfaction, enhanced self-efficacy and commitment to the profession [5]. In a seminal longitudinal study on teachers’ professional identity, Day and colleagues [12] found that the manner in which teachers were able to balance three central dimensions of their work significantly influenced their job satisfaction and psychological well-being. These dimensions included a personal dimension that related to their lives outside of the work context (e.g., spending time with family and engaging in leisurely activities), a professional dimension (i.e., their roles and responsibilities as a teacher and the expectations of school, colleagues, students and parents) and a situational dimension which entailed the context in which they worked and the extent to which the work environment was facilitative of their roles and responsibilities [12]. Teacher professional identity has been identified as a significant determinant of teachers’ motivation, job commitment and satisfaction with their work [5].

Professional identity is rooted in social identity theory [13], which proposes that group membership is an important source of pride and self-esteem. Two qualitative studies [14,15] found that professional identity and the impact of the pandemic on professional identity emerged as themes in interviews with teachers. In one of these studies, Kim and Asbury [14] found that their participants drew on characteristics they perceived as core to the teaching profession in managing remote learning and teaching. Therefore, we speculated that teachers who had a strong professional identity would manage role stress more successfully than those with a weak professional identity. Based on this assumption, we hypothesized that:

**Hypothesis** **1.** *Low levels of role ambiguity will be associated with high levels of teaching identification*.

**Hypothesis** **2.** *Low levels of role conflict will be associated with high levels of teaching identification.*

The relationship between role stress and negative indices of psychological well-being has been well established. For example, burnout [16,17], depression [18], anxiety, and hopelessness [19] have been consistently linked with role stress. In a study of university educators, Xu [17] found that role conflict was positively correlated with emotional exhaustion (r = 0.47, *p* < 0.01) and depersonalization (r = 0.31, *p* < 0.01). Similarly, Wu and colleagues [16] found that role conflict (β = 0.502, *p* < 0.01) and role ambiguity (β = 0.359, *p* < 0.01) were positively associated with burnout in a sample of construction project managers. Among a sample of healthcare workers in Qatar, Yehya and colleagues [18] found a significant positive relationship between role conflict (β = 0.35, *p* < 0.001) and role ambiguity (β = 0.37, *p* < 0.001) and depressive symptoms. In developing a new measure of work stress for correctional officers, Şenol-Durak and colleagues [19] reported positive correlations between role conflict and ambiguity (as a single measure) and depression (r = 0.40, *p* < 0.001), anxiety (r = 0.32, *p* < 0.001), and hopelessness (r = 0.22, *p* < 0.05). In a meta-analytic review of 483 studies, Greco and colleagues [20] found that professional identification and organizational identification explained a similar amount of variance in psychological well-being (42%). Based on the positive correlations identified between role stress and indices of psychological distress, we hypothesized that:

**Hypothesis** **3.** *High levels of role ambiguity will be associated with high levels of anxiety*.

**Hypothesis** **4.** *High levels of role ambiguity will be associated with high levels of hopelessness.*

**Hypothesis** **5.** *High levels of role conflict will be associated with high levels of anxiety*.

**Hypothesis** **6.** *High levels of role conflict will be associated with high levels of hopelessness.*

The term professional is often considered a desirable self-description; thus, it may be assumed that there would be positive psychological benefits when a person identifies with their profession [21]. Empirical research findings have supported the positive effect of professional identification on psychological well-being. One study distinguished between research identity and innovative identity, two aspects of teachers’ professional identification [22], and found positive relationships between each of these two types of identity and psychological well-being (r = 0.39, *p* < 0.01; r = 0.510, *p* < 0.01, respectively). In a sample of nurses in China [23], professional identity was positively associated with subjective well-being (β = 0.215, *p* < 0.001). Researchers have also found support for the mediating role of professional identification in the relationship between adverse conditions and psychological well-being. Hao and colleagues [24] found that professional identity both independently mediated the relationship between work stress and well-being (β = −0.225, 95% CI (−0.307, −0.154)) and jointly mediated this relationship in a serial relationship with psychological capital (β = −0.075, 95% CI (−0.116, −0.043)). Padmanabhanunni and Pretorius [25] used serial mediation analysis and found that teaching identification mediated both the relationship between fear of COVID-19 and teacher satisfaction (β = −0.087, *p* = 0.005) and the relationship between perceived vulnerability to disease, fear of COVID-19, and teacher satisfaction (β = −0.054, *p* = 0.004). We consequently hypothesized that:

**Hypothesis** **7.** *Low levels of role ambiguity will be associated with high levels of teaching identification, which in turn will be associated with low levels of anxiety*.

**Hypothesis** **8.** *Low levels of role ambiguity will be associated with high levels of teaching identification, which in turn will be associated with low levels of hopelessness*.

**Hypothesis** **9.** *Low levels of role conflict will be associated with high levels of teaching identification, which in turn will be associated with low levels of anxiety*.

**Hypothesis** **10.** *Low levels of role conflict will be associated with high levels of teaching identification, which in turn will be associated with low levels of hopelessness*.

The current study thus aims to examine the potential protective role that identifying with the teaching profession might play in the stress-psychological distress relationship. While the identification of cause-effect relationships related to stressors and outcomes are worthwhile endeavors, of equal importance is the identification of protective resources that makes individuals differentially vulnerable to adverse circumstances. The identification of such factors provides the basis for effective intervention that enables psychology to fulfil its mandate as a ‘helping’ profession.

## 2. Materials and Methods

### 2.1. Participants

The study participants consisted of 355 South African school teachers. Sterne [26] indicated that there were 400,000 teachers in South Africa in 2021. Our sample, thus, represents a 5.09% margin of error (95% confidence interval). A description of the demographics of the sample is presented in Table 1. Most participants were women (76.6%), resided in the Western Cape (82.3%) and in an urban area (61.7%) and taught at the primary school level (61.1%). The mean age of the sample was 41.9 years (±12.42), and the mean number of years in the teaching profession was 15.7 years (±11.75).

### 2.2. Instruments

Participants completed a demographic survey that requested them to indicate their gender, the province in which they reside, their age, and other relevant demographic variables. In addition, participants completed the following questionnaires: the Role Orientation Questionnaire (ROQ) [8], the Professional Identity Scale (PIS) [27], the trait scale of the State-Trait Anxiety Inventory (STAI-T) [28], and the Beck Hopelessness Scale (BHS) [29].

The ROQ is a 14-item measure of perceived role stress in the work environment, which is measured on a 6-point Likert scale ranging from “Definitely not true of my job” (1) to “Definitely true of my job” (6). Eight items measure the degree of incongruity experienced regarding role expectations or role conflict (RC; e.g., “I receive incompatible requests from two or more people”). Six items measure perceived lack of clarity regarding role expectations or role ambiguity (RA; e.g., “Explanation is clear of what has to be done”). In the original study, the authors reported internal consistency coefficients of 0.82 (RC) and 0.78 (RA) for the ROQ [8]. The relationships between role conflict and role ambiguity and managerial practices, leader behavior, job satisfaction, anxiety, and turnover intention provide evidence of the tool’s validity [8]. More recent studies have reported similarly satisfactory estimates of internal consistency for the ROQ, for example, Orgambídez and Almeida: ω = 0.92 and 0.85 for RA and RC, respectively [30], and Novriansa and Riyanto: composite reliability = 0.80 and 0.79 for RA and RC, respectively [31]. A 21-nation study of role conflict and role ambiguity reported alpha coefficients of 0.54 and 0.87, respectively, for the use of the ROQ scale with South African participants [32].

The PIS is a generic, as opposed to teacher-specific, measure of the extent to which an individual identifies with a particular group. Based on social identity theory, the PIS consists of 10 items measured on a 5-point scale ranging from 1 (Never) to 5 (Very often). An example of a generic item from the scale is “I am a person who feels strong ties with the… group”. When using the PIS for teachers, the wording of this item is changed to “I am a person who feels strong ties with the teaching profession”. In the original study, the authors reported an estimate of internal consistency of 0.71 for the PIS [8]. Validity was established by comparing the PIS scores of those who expressed positive views about their group with those who expressed negative views. In more recent studies, similarly satisfactory reliability coefficients were reported; for example, Zeng et al.: α = 0.82 [33] and Sun et al.: α = 0.82 [34]. In a South African application of the scale, Padmanabhanunni and Pretorius [25] reported estimates of internal consistency exceeding 0.70 (α = 0.85, ω = 0.83).

The STAI-T is a measure of trait anxiety that consists of 20 items scored on a 4-point scale ranging from 1 (Almost never) to 4 (Almost always). An example item of the STAI-T is “I worry too much about things that really doesn’t matter”. Spielberger [28] reported test-retest reliabilities ranging from 0.73 to 0.86. Validity was demonstrated through significant relationships with other measures of anxiety and the scale’s ability to distinguish between control samples and samples in highly stressful situations. A recent meta-analysis reported that the STAI-T demonstrated high reliability (α = 0.87–0.93) and differentiated between the general population and people with anxiety [35]. Similarly, recent studies in non-English-speaking countries reported highly satisfactory reliability coefficients for the STAI-T, for example, Iran: α = 0.86 [36]; Italy: α = 0.92 [37]. Padmanabhannuni and colleagues [38] reported very satisfactory estimates of internal consistency for the STAI-T when used with South African respondents (α = 0.91, ω = 0.91).

The BHS is a measure of hopelessness or negative expectations about the future. It consists of 20 items scored on a true-false rating scale. An example of an item from the BHS is “All I can see ahead of me is unpleasantness rather than pleasantness”. Beck and colleagues [29] reported a satisfactory estimate of internal consistency for the BHS (KR-20 = 0.93). They also found that scores on the BHS correlated significantly with clinical ratings of hopelessness, as well as other measures of hopelessness. The BHS has been used in a variety of countries and with a variety of populations, and satisfactory reliability coefficients have been reported in all studies of the instrument; for example, Spain, suicide attempters: α = 0.98 [39]; Spain, elderly respondents: α = 0.89 [40]; China, rural suicide attempters: α = 0.95 [41]. South African studies have also reported satisfactory estimates of internal consistency in a university student sample: α = 0.88, ω = 0.88 [42]; and in a sample of school teachers: α = 0.89, ω = 0.89 [38].

### 2.3. Procedure

The study used a cross-sectional survey design with a convenience sample. Google Forms was used to develop an electronic version of the questionnaires. School teachers from across South Africa were invited to participate through a link that was posted on several teacher Facebook groups. In addition, the link was provided by email to teachers who requested it. Data collection took place during the second wave of the pandemic (May–July 2021).

### 2.4. Ethics

Ethical approval for the study was granted by the Humanities and Social Sciences Ethics Committee of the University of the Western Cape (ethics reference number: HS21/3/8). Teachers participated anonymously and provided informed consent.

### 2.5. Data Analysis

Descriptive statistics, reliability coefficients, and intercorrelations were obtained using IBM SPSS Statistics for Windows (version 28; IBM Corp., Armonk, NY, USA). Structural equation modelling with IBM SPSS Amos (version 28; IBM Corp.) was used to examine the potential mediating role of teacher identification in the relationship between role stress and psychological distress. The significance of direct and indirect effects within the model was evaluated using bootstrapped confidence intervals (95% CI).

## 3. Results

The descriptive statistics (means and standard deviations), internal consistency coefficients (Cronbach’s alpha and McDonald’s omega), and intercorrelations between variables are reported in Table 2. The reliabilities of all scales (α and ω) are very satisfactory, and all exceed the recommended level of 0.70 [43].

As illustrated in Table 2, role stress was negatively correlated with teaching identification (RC: r353 = −0.19, 95% CI [(0.29, −0.09), *p* < 0.001; RA: r353 = −0.40, 95% CI (−0.48, −0.31), *p* < 0.001) and positively correlated with anxiety (RC: r353 = 0.27, 95% CI (0.16, 0.36), *p* < 0.001; RA: r353 = 0.22, 95% CI (0.11, 0.31), *p* < 0.001). Thus, high levels of teaching identification are associated with low levels of role stress, whereas high levels of role stress are associated with high levels of anxiety and hopelessness. Teaching identification was negatively related to anxiety (r353 = −0.32, 95% CI (−0.41, −0.23), *p* < 0.001) and hopelessness (r353 = −0.39, 95% CI (−0.48, −0.30), *p* < 0.001). Thus, high levels of teaching identification are associated with low levels of anxiety and hopelessness.

There were several significant gender differences and differences between rural and urban teachers. Women reported significantly higher levels of anxiety (mean = 46.03, SD = 10.46, t = 3.76, *p* < 0.001) and hopelessness (mean = 6.16, SD = 5.08, t = 3.02, *p* = 0.003) than men (anxiety: mean = 41.24, SD = 8.89; hopelessness: mean = 4.30, SD = 4.14). Urban teachers reported higher levels of anxiety (mean = 46.29, SD = 10.29, t = 3.21, *p* = 0.001) than rural teachers (mean = 42.73, SD = 9.98). There was also a significant negative relationship between age and anxiety (r353 = −0.19, *p* < 0.001).

**Figure 1:**
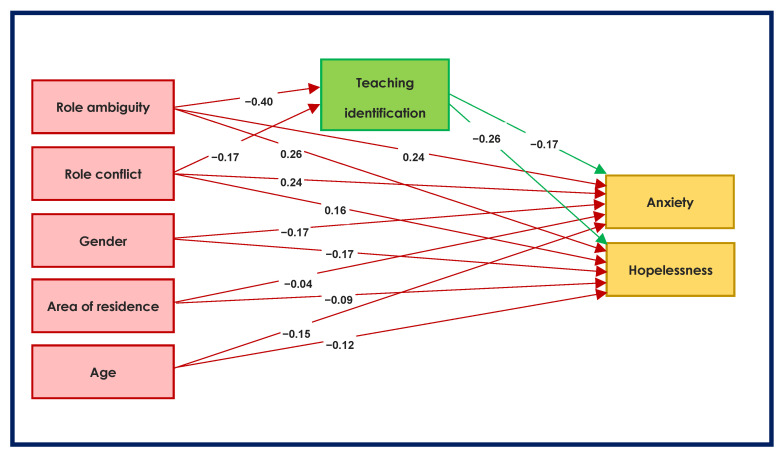
Structural equation model of the relationship between role stress, teaching identification and psychological distress. Note. Regression weights are standardized. All paths involving independent variables, mediators and dependent variables are significant *p* < 0.01.

The structural equation model that was used to examine the direct and indirect effects of role stress is presented in Figure 1. In this model, role conflict and role ambiguity are the predictors, anxiety and hopelessness are the dependent variables, and teaching identification is the potential mediator. Given the significant gender, rural-urban and age associations with hopelessness and anxiety, these demographics were controlled by adding them as covariates in the model.

The direct and indirect effects resulting from the structural equation model are presented in Table 3. All direct and indirect effects were significant. Thus, all the study hypotheses were supported by the mediation analyses.

Specifically, Table 3 confirms the hypotheses that:

H1: Low levels of role ambiguity were associated with high levels of teaching identification (β = −0.395, 95% CI (−0.472, −0.308), *p* < 0.001).

H2: Low levels of role conflict were associated with high levels of teaching identification (β = −0.173, 95% CI (−0.254, −0.094), *p* < 0.001).

H3: High levels of role ambiguity were associated with high levels of anxiety (β = 0.266, 95% CI (0.178, 0.342), *p* < 0.001).

H4: High levels of role ambiguity were associated with high levels of hopelessness (β = 0.261, 95% CI (0.186, 0.237), *p* < 0.001).

H5: High levels of role conflict were associated with high levels of anxiety (β = 0.223, 95% CI (0.138, 0.300), *p* < 0.001).

H6: High levels of role conflict were associated with high levels of hopelessness (β = 0.159, 95% CI (0.083, 0.232), *p* < 0.001).

H7: Low levels of role ambiguity were associated with high levels of teaching identification, which in turn were associated with low levels of anxiety (β = 0.069, 95% CI (0.063, 0.203), *p* = 0.001).

H8: Low levels of role ambiguity were associated with high levels of teaching identification, which in turn were associated with low levels of hopelessness (β = 0.101, 95% CI (0.055, 0.130), *p* < 0.001).

H9: Low levels of role conflict were associated with high levels of teaching identification, which in turn were associated with low levels of anxiety (β = 0.030, 95% CI (0.015, 0.071), *p* = 0.001).

H10: Low levels of role conflict were associated with high levels of teaching identification, which in turn were associated with low levels of hopelessness (β = 0.044, 95% CI (0.013, 0.046), *p* < 0.001).

## 4. Discussion

The current study examined the relationship between role stress and negative indices of psychological well-being, as well as the potential mediating role of professional identification in this relationship. The study findings confirm the positive relationship between role stress and psychological distress previously reported in the literature [16,17,18,19]. Specifically, the results indicate that high levels of role ambiguity and conflict were associated with high levels of anxiety and hopelessness. In a study of medical doctors, Abbasi [44] found that role stress accounted for 48% of participants’ variance in health (as measured by the General Health Questionnaire). The same study found that transformational leadership moderated the relationship between role stress and health. Similarly, supervisor support has been found to moderate the relationship between post-traumatic stress (PTSD) and turnover intention among nurses [45]. These studies underscore the important role of leadership in alleviating the impact of adverse work conditions. Strengthening supervisor support at the school level for teachers during the pandemic should be considered an important policy imperative. The sudden transition to remote teaching and learning has inevitably led to increased role ambiguity and role conflict among teachers. However, Kraft and colleagues [9] aptly observed that a crisis like the COVID-19 pandemic can also be a catalyst for change. In this regard, national and provincial departments of education have an important role to play in supporting teachers and also ensuring regular and unambiguous communication. In a qualitative study of teacher experiences during the pandemic, Reich and colleagues [15] found that some interviewees reported being extremely frustrated by school, district, or state changes in policy or expectations. To respond to these frustrations, they recommend that education departments provide “clearly communicated, simply designed, and consistently maintained expectations” to teachers [15] (p. 19).

A second finding of the current study is that teaching identification was negatively associated with role stress and indices of psychological distress. Thus, high levels of teaching identification were associated with low levels of role stress, anxiety, and hopelessness. No prior studies have explicitly focused on the relationship between role stress and professional identification. In a qualitative study, Kim and Asbury [14] identified teacher identity as a core variable that emerged from interviews in which teachers explained how they had coped during the pandemic. Therefore, teachers with a strong teaching identity may be less likely than their colleagues to experience their work context during the pandemic as conflictual or ambiguous. In the present study, teaching identification also had direct effects on anxiety and hopelessness. This finding supports previous research findings that have linked professional identification to positive health outcomes [20,22,23]. It is also consistent with the health-sustaining hypothesis [46], which proposes that a variable that is presumed to moderate or mediate the effects of a stressor on health outcomes may also directly affect the outcome, irrespective of the level of the stressor. In other words, even in the absence of stressors, having a strong teacher identity is beneficial for health.

Third, the current study findings indicate that teaching identification mediated the relationship between role stress and indices of psychological distress. Low levels of role ambiguity and conflict were associated with high levels of teaching identification, which in turn was associated with low levels of anxiety and hopelessness. This result supports prior findings related to the mediating/moderating role of teaching identification [24,25]. Teachers with a strong professional identity may see their role as a calling rather than a job. Being a teacher is a matter of pride that engenders a positive self-concept [10]. Therefore, these teachers may be determined to continue their teaching mandate despite the pandemic. In a qualitative study, Kim and Asbury [14] identified a theme of “making it work”, which emerged from their interviews in reference to the ways in which teachers have adjusted their thinking and behavior to continue providing teaching remotely. Determined to “make it work”, these teachers focused on the task at hand and did not experience their work context as conflictual or ambiguous; in turn, this mindset had a positive impact on their psychological well-being.

## 5. Implications

The findings of the current study highlight the critical importance of professional identity, specifically teacher identity. In the context of a global devaluing of the teaching profession [47], this is an important finding that should provide a wake-up call to government officials, teaching unions, and teaching associations. Focusing on something as basic as improvements to teacher remuneration could affect the extent to which the profession is valued by society. Falla [48] contends that implementing policies such as teacher sabbaticals and revisions to hiring practices and evaluation processes could help to restore the valuation of the teaching profession. Teaching identity formation starts at the university level as students prepare to become teachers. It is important at this stage that the values and attitudes consistent with a strong professional identity are formally inculcated as part of the curriculum.

## 6. Limitations

The study has certain limitations. First, we used a cross-sectional survey design, which limits the extent to which causal relationships can be conclusively identified. Future studies could use a longitudinal design to determine causality. We also used a convenience sample, which limits the generalization of the study findings. Finally, we used self-report measures, which are subject to social desirability bias. However, our findings are consistent with those reported in the existing literature. The method of data collection, which was unavoidable due to COVID-19 restrictions, may also have impacted the composition of the sample as only teachers with access to technology or who are competent in the use of technology may have responded. However, it has been reported that 99.9% of the South African population had access to mobile networks, and smartphone penetration was 91.2% as of 2021 [49].

## 7. Conclusions

The relationship between stress and negative outcomes has been well documented. However, in addition to understanding the cause-effect relationship, it is important to examine the protective factors that moderate or mediate this cause-effect relationship. The current study focuses on professional identification as one such variable that intervenes in the cause-effect relationship and found that low levels of role stress were associated with high levels of teacher identification, which in turn were associated with low levels of anxiety and hopelessness. The results have strong implications for the role of leadership and supervisor support in protecting teachers’ health and well-being, as well as enhancing the societal value of teaching as a profession.

## Figures and Tables

**Table 1:** Demographic description of the sample.

Variable	Categories	*n*	%
Gender	Male	83	23.1
	Female	272	76.6
	Non-binary	1	0.3
Province	Eastern Cape	12	3.4
	Western Cape	292	82.3
	Gauteng	31	8.7
	KwaZulu Natal	10	2.8
	Mpumalanga	2	0.6
	North West	3	0.8
	Limpopo	2	0.6
	Free State	3	0.8
Area of residence	Rural	136	38.3
	Urban	219	61.7
Grade teaching	Pre-primary	14	3.9
	Primary	217	61.1
	Secondary	122	34.4
	Learning support	2	0.6
Age	Mean (SD)	41.9 (12.42)	
Years teaching	Mean (SD)	15.7 (11.75)	

**Table 2:** Descriptive statistics, reliabilities, and intercorrelations between study variables.

Variable	1	2	3	4	5
1. Role conflict	-	(−0.06, 0.14)	(−0.29, −0.09)	(0.16, 0.36)	(0.11, 0.31)
2. Role ambiguity	0.04	-	(−0.48, −0.31)	(0.25, 0.43)	(0.28, 0.46)
3. Teaching identification	−0.19 ***	−0.40 ***	-	(−0.41, −0.23)	(−0.48, −0.30)
4. Anxiety	0.27 ***	0.34 ***	−0.32 ***	-	
5. Hopelessness	0.22 ***	0.37 ***	−0.39 ***	0.62 ***	-
Mean	30.4	14.7	40.1	44.9	5.73
SD	8.2	5.7	6.9	10.3	4.9
Alpha	0.83	0.83	0.85	0.91	0.89
Omega	0.83	0.83	0.83	0.91	0.89

Note. Correlation coefficients are below the diagonal, and 95% confidence intervals are above the diagonal. *** *p* < 0.001.

**Table 3:** Direct and indirect effects of role stress.

Effect	Beta	SE	β	95% CI	*p*
Direct effects					
Role ambiguity → Teacher identification^H1^	−0.484	0.063	−0.395	[−0.472, −0.308]	0.001
Role conflict → Teacher identification^H2^	−0.146	0.041	−0.173	[−0.254, −0.094]	0.001
Role ambiguity → Anxiety^H3^	0.432	0.091	0.241	[0.153, 0.318]	0.002
Role ambiguity → Hopelessness^H4^	0.228	0.041	0.260	[0.183, 0.332]	0.001
Role conflict → Anxiety^H5^	0.290	0.061	0.235	[0.152, 0.312]	0.001
Role conflict → Hopelessness^H6^	0.099	0.027	0.164	[0.091, 0.234]	0.001
Teaching identification → Anxiety	−0.248	0.079	−0.169	[−0.248, −0.075]	0.001
Teaching identification → Hopelessness	−0.189	0.040	−0.264	[−0.349, −0.173]	0.003
Indirect effects					
Role ambiguity → Teaching identification → Anxiety^H7^	0.120	0.041	0.067	[0.058, 0.196]	0.001
Role ambiguity → Teaching identification → Hopelessness^H8^	0.091	0.022	0.104	[0.058, 0.131]	<0.001
Role conflict → Teaching identification → Anxiety^H9^	0.036	0.016	0.029	[0.015, 0.069]	0.001
Role conflict → Teaching identification → Hopelessness^H10^	0.028	0.010	0.046	[0.014, 0.047]	<0.001

Note. Superscript notation refers to hypothesis numbering.

## Data Availability

The raw data supporting the conclusions of this article will be made available by the authors without undue reservation.
